# Precautions against Fire in Hospitals and Kindred Institutions

**Published:** 1904-06-18

**Authors:** 


### PRECAUTIONS AGAINST FIRE IN HOSPITALS AND KINDRED INSTITUTIONS.

The consequences of fire spreading through any of our hospitals, where several hundreds of sick people are located, many of them unable to help themselves in the panic which is sure to accompany a fire, would be so terrible that we have made inquiries at some of the large London and provincial hospitals regarding the means they had adopted to secure the safety of their patients and the protection of the building in the event of an outbreak; and we hereby wish to thank the officials for their courteous, and in some cases very full answers to our inquiries. The question naturally divides itself into four heads. 1. The prevention of fire. 2. The safety of the patients. 3. The means at hand inside the building which the resident staff can bring into use immediately. And 4. The means which exist outside the building for coping with the fire when it has got firm hold, and when it may be presumed the patients have been removed to a place of safety.

1. *As regards Prevention of Fire.*—One of our correspondents remarks that the danger of outbreak of fire is much greater in the nurses' quarters than in the wards, and beyond doubt this is so. Where gas is the illuminant the use of inflammable curtains and blinds is a common source of danger, and their use should therefore be abolished. Outside the room there should be a controlling tap which shuts off the gas from the corridor or wing where the nurses' room or ward is situated, and where no such tap exists nurses should be instructed how to stop a leaking pipe by soap or other means. Chimneys should be carefully and frequently swept to prevent the accumulation of soot, which has a tendency to "cake" and fall down in burning masses. Fenders should be made of fixed kerb stones, high enough and wide enough to contain such soot or live coal which may fall on the hearth, and the hearthstone should have a thick layer of concrete underneath. Carrying fire from one part of the building to another should be strictly forbidden, and only safety matches should be allowed and these should be in the nurse's charge. Where ward floors are of oak, teak, or maple, a mixture of wax and turpentine is used for polishing them, and this mixture should be prepared outside the wards and kept away from all source of danger, but it is better to mix only as much as will be required at the time and all articles used in polishing the floors should be kept with equal care. Coal boxes are best made of metal, and special care must be taken in the disposal of hot ashes, as they have caused many a fire. Electric mains should be carefully isolated. So should steam mains, and hot-air flues especially should be constructed with the greatest care and should be often examined. Hot-water or steam radiators should be made to swing outwards, so that they may be frequently cleaned from dust, or fluff, or from the accidental lurking of a piece of rag. Where smoking is allowed, the number of pipes in use should be known, and they should be given up by the patients at a fixed hour every evening. No excuse should be held valid for the employment of a spirit or paraffin stove or paraffin lamp in the nurses' rooms or elsewhere, and the use of candles should be prohibited.

2. *The Safety of the Patients.*—It is an arguable point whether a hospital should ever be more than one story high, quite irrespective of danger from fire, and if this form of construction were universal, the safety of the patients might be ensured by the simplest means; but unfortunately such construction is absolutely impossible in the limited areas at disposal in our large towns, and consequently the buildings are run up to three, four, or perhaps five stories in height. We are now in the age of electric power. Lifts are almost invariably used, and they obviate the drawbacks formerly inseparable from high buildings. It is even probable that, in London at anyrate, the higher wards receive purer air, and hence may be more sanitary, than the lower ones. At present we must deal only with the fire aspect of the subject, and we find that outside staircases, or balconies, or both, are practically universal in these high hospitals. So far so good, and in a patient able to walk without help, escape is certain, as these staircases are always at the opposite end of the ward to the main staircase, and one or other is sure to be available. But let us suppose the case of a patient, who from some cause could not walk. How is he to be got down a staircase often not more than 2 feet 6 inches or 3 feet wide? Are sufficient stretchers at command? and are nurses trained for this duty? In view of this difficulty, which would certainly be met with in the first serious fire at any of our large hospitals, we are inclined to think that safety lifts would be much more reliable than safety staircases, or the lifts might be fitted up as adjuncts to the staircases. Against this it would be possible to plead the extra expense involved; but when £300,000 may be spent in the erection of a hospital, an additional thousand or so on the lifts need not be too seriously considered if it could be proved that the benefit would be commensurate with the expense. Bedsteads are now made with large rubber castors and can be easily moved from one part of the ward to another without disturbing the patient, and several wheeled chairs should form part of the equipment of every ward. One section of the nurses should be carefully and regularly drilled in the use of lifts, staircases, canvas shoots and stretchers. "Dummy" patients should be used, and such section of the staff should have nothing to do with the fire brigade part, the duties of each section being clearly defined, and instructions carefully given. The latter would include the best means of preventing the inhalation of smoke.

3. *The Means at Hand for Immediately Subduing a Fire.* These include buckets, extinguishers of various kinds, corridor engines, grenades and hydrants; and many of our hospitals possess all these; but whether they exist in sufficient numbers we are unable to form an opinion. It is curious that none of our correspondents refer to the nests of buckets. These are usually six in number, and are contained in a tank full of water. As one bucket is drawn out the next one is immediately filled. The advantages which these possess are that they occupy but little space, do not accummulate dust, do not often need seeing to, are not unseemly in the neatest ward, and may be easily handled by any nurse. The extincteurs in common use contain various chemical substances (carbonic acid, tungstate of soda, etc ), and when they are in proper working order are very useful, as they do more work than many times the volume of plain water. We believe a new form has lately been placed on the market which remains good for a very long time. The value of the internal hydrant will depend upon its being always ready with sufficient length of hose attached to reach to the furthest point of the ward. Much of the usefulness of these appliances will be lost unless the fire brigade section of the staff is drilled to such a point that on the alarm of fire each member will perform his or her part almost automatically. No man is a soldier until he obeys the word of command without having to reflect on its meaning; and so the sound of the fire alarm should send everyone to his or her post. We find that in some hospitals this drill takes place once a week, in others once a fortnight, and in others once a month or even less frequently, and in a few no drilling at all takes place. One of our correspondents justifies this omission on the ground that the first duty of the nurse is to her patients. And so it is; but it is not her only duty, and as already said we believe that this drill should be frequent, and that it should never be relaxed. It is useless having a hose always ready if the water is turned on with so many twists in the hose that the water will not run through it; and this is one of the commonest mistakes of the undrilled. A ward of ordinary size, say for about twenty patients, should possess at least one dozen buckets, two corridor engines, and two hydrants. The buckets should be placed on the floor, or within three feet of the floor. The corridor engines should be in some place from which they can be easily wheeled out. The hoses should be always fixed to the hydrants, or should have the patent instantaneously-fixed couplings. Nothing is so foolish as to place fire buckets or fire engines out of sight.

While the members of the safety brigade are getting the patients out of the block in which the fire has broken out, the fire brigade section will be using the means at hand for putting the fire out. A few buckets of water and a corridor engine, if at hand and in working order, will often do this; and in few things is prompt action more likely to be met with success than in the beginning of a fire, but as already said the action must be instantaneous, almost automatic, and free from the least confusion; hence long and careful drilling is the only means of making these points certainties when the day of battle comes. Once a week is not too often for these drills, and as many of the staff as can be spared should attend them; and in addition to the drills there should be fire alarms at uncertain intervals, and this alarm should be the signal for everyone who could be spared from other blocks assembling in the block in which the fire is supposed to exist. The ward sisters would decide on this number.

One of the superior staff should have charge of the drill classes, and another should direct the safety brigade, but each of these should know the duties of the other. The former would be on the spot immediately on the sounding of the fire alarm, and the latter would send additional help from other blocks as soon as she had satisfied herself that the patients were safe. The fire brigade staff would be divided into three sections: the first having charge of the buckets, the second of the corridor engines, and the third of the hoses. One nurse or wardmaid from the bucket section should fill all the baths, so that no time would be lost in refilling the buckets, which loss of time would certainly happen if they had to be refilled from taps. All the fire brigade staff should be trained in all parts of the service, but each ward should have placed in some conspicuous part the names of the nurses and wardmaids who would see to the buckets, corridor engines, and hoses respectively, for the month, or quarter, or half-year as the rule might be. By this division of labour all confusion ought to be avoided, and all sections would concentrate their appliances on the fire as soon as possible after the outbreak. If this has been on or near the floor the buckets may be sufficient before the hose is brought into use, but if it be a few feet high the corridor engines would be most useful and the buckets would be used for keeping the engines full of water. The domestic staff should be equally trained with the nursing staff in the use of all fire appliances, and should have their stations and duties as accurately stated.

It cannot be denied that these fire alarms and fire practices disturb the arrangements of the wards or blocks, and hence they are not always popular with the ward sisters; but the advantages, nay the absolute necessity, of these fire alarms are so patent that all temporary inconveniences must be borne as well as possible. As regards the weekly drills they can be done very well out of doors provided hoses, engines, and tanks are conveniently placed, and at all these drills the instructor should in every practice check the time in which the hoses have been got ready, the corridor engines playing, and the buckets in use. The committee would do well to encourage this promptness by granting prizes or extra leave of absence to the staff of the block which came out first on the list. When the drill is in the ward, or when the fire alarm is sounded, the water must be turned on, but the nozzles of the hoses may be placed out of the windows, and the buckets may be filled from a fixed bath and emptied into a portable bath. Dry fire-drills are delusions. No one can tell whether the hoses are properly uncoiled, the corridor engine pumps in order, or the buckets filled quickly enough.

*The Means which Exist Outside the Building.*—These are the external hydrants and the pressure in them should be always sufficient to take the water clear over the highest roof. This would be the duty of the outdoor male staff, and in the meantime the local town brigade would have been summoned. Ladders and other fireman's appliances would form part of the hospital armamentarium. All the hospitals from which we received answers are in telephonic communication with the fire stations or are within very short distances of the local fire alarm. We may feel confident that the possibility of fire has not been overlooked by our hospital officials, but it may very well be that in some institutions the means at immediate command and the amount of drilling of the staff are hardly up to the standard which we should like to see universally attained.

In the modern hospital construction is for the most part fireproof, that is, it is composed of material which will not readily catch fire, and so time would be ample to permit of the removal of the patients, but it by no means follows that the building will be saved. Where practicable fireproof doors should be placed, as at least they have the effect of stopping the draught for a time and thus check the passage of smoke. The fire-alarm signal is usually a steam whistle, buzzer, or syren. During the day it is easily reached, but it might be difficult for the night staff to get at it. This difficulty must not be lost sight of, as a fire during the night in a hospital would be even more apalling than during the day and there would be fewer nurses at immediate command.

Mr. Long, of the Stafford County Asylum at Cheddington, has lately issued an admirable little book called "Sparks" for the instruction of asylum nurses and attendants in their fire duties. It contains a great deal of information, carefully arranged and clearly conveyed, and we strongly recommend it not only to asylum officials but to hospital nurses. With a few sentences omitted here and there which apply to asylums only it is just such a book as the instructor of a hospital fire brigade should have at his finger ends, and by insisting on the nurses studying it the labour of personal instruction would be considerably lessened. It is published by W. H. Eaton, Derby Street, Leek.

